# Animal Models for Studying Stone Disease

**DOI:** 10.3390/diagnostics10070490

**Published:** 2020-07-18

**Authors:** Szu-Ju Chen, Kun-Yuan Chiu, Huey-Yi Chen, Wei-Yong Lin, Yung-Hsiang Chen, Wen-Chi Chen

**Affiliations:** 1 Division of Urology, Department of Surgery, Taichung Veterans General Hospital, Taichung 407204, Taiwan; u99001099@gap.kmu.edu.tw (S.-J.C.); chiu37782002@yahoo.com (K.-Y.C.); 2 Departments of Obstetrics and Gynecology, Medical Research, and Urology, China Medical University Hospital, Taichung 404332, Taiwan; d888208@ms45.hinet.net; 3 Graduate Institute of Integrated Medicine, College of Chinese Medicine, China Medical University, Taichung 404333, Taiwan; linwy@mail.cmu.edu.tw; 4 Department of Psychology, College of Medical and Health Science, Asia University, Taichung 413305, Taiwan

**Keywords:** stone disease, calcium oxalate, *Drosophila melanogaster*, animal models

## Abstract

Animals have stone disease too. There are several animal models for the research of human stone disease. Rodents are the most frequently used for stone research, although they are not prone to forming crystals in the kidneys. Ethylene glycol (EG), sodium oxalate and l-hydroxyproline are common lithogenic agents. Dogs and pigs were also reported as a study animal for stone disease. However, the breeding costs and body size are too high. The most-used genetic study animal for stone disease was the mouse, but it was high-cost. Calcium oxalate (CaOx) crystals can also be light microscopically observed in the Malphigian tubules of *Drosophila melanogaster*, induced by adding EG to the food. Genetic studies of flies can be done by cross-breeding, and this has a lower cost than using mice. The fly model also has several advantages, including minimal breeding equipment, the fact that it is easier to reach larger numbers in a short time with flies, that crystals can be observed under microscopy, and that they allow genetic study. We suggest the fly will be an ideal animal model for stone research in the future.

## 1. Introduction

Humans are not the only animal with urolithiasis. Robbinson et al. reviewed PubMed for various animal species, and found a total of 919 citations regarding affected urolithiasis [1]. The reported cases of stone disease in two- or four-legged animals included non-mammals, and mammals such as dogs, cats, birds and turtles. In Taiwan, an early report of stone disease has been achieved not only in humans, but also in monkeys (*Macaca cyclopis*) [2]. Stone disease is a universal phenomenon throughout the animal kingdoms; therefore, the use of animal models to study the disease may have the advantage of similar pathogeneses, and may also have benefits both for humans and animals [3]. In this review, we searched the current types of animal models used in the stone disease research literature on PubMed, in order to share with the readers.

## 2. Animal Study for Stone Disease in Taiwan

In Taiwan, the first documented use of rats as a study animal for stone disease was reported by Lee et al. in 1991 [4]. They used rats as study animals, and ethylene glycol (EG) as the lithogenic agent to characterize the calcium oxalate (CaOx) crystals in the kidney. In 1996, adult Sprague-Dawley rats were used to study the effects of sex hormones on stone disease [5]. The lithogenic agent used was 0.5% EG in the feeding diet. Their results demonstrated that testosterone enhances urolithiasis, and estrogen inhibits CaOx formation [5]. Thereafter, many following urolithiasis researchers also used the rat as a study animal [6,7,8,9]. Huang et al. added 0.75% EG to the drinking water to study the rats’ nephrolithiasis, and found that free radicals occurred mainly in the blood during stone formation [6]. They further studied the effect of EG in the rat model, and found that free radicals are responsible for oxalate toxicity [9].

The rat model was also used to investigate or screen potential agents for preventing urolithiasis. Tsai et al. investigated the effects of a stone-preventing traditional Chinese herbal formula, Wulingsan (WLS), using an EG-induced nephrocalcinosis rat model [7]. The results indicated that WLS effectively inhibited calcium oxalate crystals from being deposited in the rat’s kidney. They also used the same model to study Zhulingtang for the effect of stone prophylaxis [10]. Lin et al. used this model to study extracts of *Flos carthami* for stone prevention [11]. *Flos carthami* is a traditional Chinese herb drug, which is active in enhancing blood flow and reducing blood stasis. The result from their study indicated that *Flos carthami* has the ability to inhibit the crystal deposition of CaOx in EG-fed rats.

The rat model was also used in a proteomic study of rat kidneys deposited in by CaOx crystals [12]. The renal cortex was harvested from EG-induced male Sprague-Dawley rat kidneys, and from controls, to compare the protein profile by means of matrix-assisted laser desorption/ionization time-of-flight mass spectrometry. The results found a reduced amount of albumin in the EG-treated group. Chen et al. used the rat model to study the expression of alanine-glyoxylate aminotransferase 2 in EG-induced kidneys [13].

In an endemic era of melamine-contaminated milk causing stone disease, many studies have focused on the major complication, i.e., stone disease [14,15]. Chen et al. studied the effects of melamine and cyanuric acid on kidney stones in rat [16]. During acute intoxication, melamine and cyanuric acid injured the proximal tubular cells and subsequently blocked the distal tubules. The results from their study revealed that crystals were distributed in both the proximal and distal tubules in rats. Chen et al. used polarized microscopy, scanning electron microscopy and energy dispersive X-ray spectroscopy microanalysis to study melamine-induced stone disease in a fly model [17]. They found stone compositions induced by melamine, not only by itself but also mixed with uric acid and CaOx.

Using the Malpighian tubules of *Drosophila melanogaster* is part of a powerful new animal model for studying stone disease. There is a world-leading team studying stone disease using *Drosophila melanogaster* in Taiwan. The composition of the crystal in the Malpighian tubules was identified by scanning electron microscopy, and was easily observed under polarized microscopy [18]. This novel animal model has been used to test the effect of potassium citrate, a clinical CaOx-preventive drug, and as the positive control in a further study. This model has been further used to screen some potential antilithic or lithogenic agents, such as commercial drinks, herbal medicines, cola, ractopamide and hydroxycitric acid [17,19,20,21,22,23].

## 3. Animal as Study Model

### 3.1. Ureter Peristalsis

The study of the peristalsis properties of the intravesical ureter isolated form a pig has been performed by Hernandez et al. [24]. They found that noradrenaline, via both α- and β-adrenoceptors, modulates both the basal tone and phasic activity of peristalsis in the pig’s intravesical ureter. Among α-1, β-1 and β-2 adrenoceptor blockers can inhibit ureteral peristalsis and the resulting dilatation of the ureter. Dilation of the ureteral lumen may theoretically facilitate stone passage. The results provide a scientific basis for the medical expulsion therapy for ureteral stones. Various types of α-blocker have been proposed to facilitate ureteral stone passage clinically [25].

A human ureter, obtained from a surgery for renal carcinoma of a renal pelvis, was also available for the studying of ureteral peristalsis. Sigala et al. used a human ureter to isolated RNA, and performed a semi-quantitative real-time polymerase chain reaction (RT-PCR) to analyze α1-adrenoceptor subtypes [26]. They used saturation-binding curves, and selective antagonists to complete the binding curves, as a study model for the protein expression of the α1-adrenoceptor. They found that the human ureter was endowed with each α1-adrenoceptor subtype, including α1A, α1B and α1D. Rajpathy et al. also studied ureter peristalsis in the human-donated ureters [27]. The excess ureters were obtained from liver donors who underwent ureteroneocystostomy. The effect of tamsulosin on the ureteric peristalsis was studied in order to evaluate its mechanism of medical expulsion therapy. They found that tamsulosin inhibited the peristaltic activity of the human ureter.

Our clinical trial is based on an animal study, for demonstrating the potential effectors of ureteral peristalsis. However, in vivo study is difficult to perform for many reasons, such as the observation method, animal ethics, anesthesia, etc. Wu et al. used an isolated porcine ureter to test the effect of drugs on ureteral peristalsis [28]. The isolated porcine ureter was obtained from a pig sample, and was free of charge, with no anesthetic or animal ethics problems. The harvested ureter should be stored in a preoxygenated Kreb’s solution (pH 7.4) before experiment. During the experiment, the ureter was suspended in an organ bath and connected to a tension transducer. The peristalsis of the ureter can be recorded, and the effects of different tested agents can be compared. The results found that α-blockers, such as doxazosin, tamsulosin and terazosin, had an inhibitory effect on proximal ureter peristalsis. Wu et al. followed this model to test the effects of the Chinese herb *Flos carthami*, and compared these with the effects of α1-adrenergic antagonists on proximal ureteral peristalsis [28]. Therefore, the porcine ureter appears to have many advantages in studying potential drugs-effect on peristalsis.

### 3.2. Dog

Dogs were used for animal model studies of stone disease in 1980 [29]. A total of 60 surgically implanted renal pelvic stone dogs were experimentally treated with high-energy shock waves. The results indicated that the shock wave was a promising form of treatment, which disintegrated the renal stone in a successful and less invasive way. The procedure of dog renal pelvic stone implantation was done in several steps, from ligation of the distal ureter, to causing pelvic dilation, to stone implantation, to distal ureteral reimplantation, to shock wave treatment, and finally to the pathology harvest [30]. However, the ethical issue for animal research has been raised for decades. In view of animal ethics flexibility [31,32], one should justify the benefit of the study in relation to animal harm, especially with regards to such a beloved animal. The dog model for stone research is therefore limited, and less prevalent in the literature.

### 3.3. Rat

**Figure 1:**
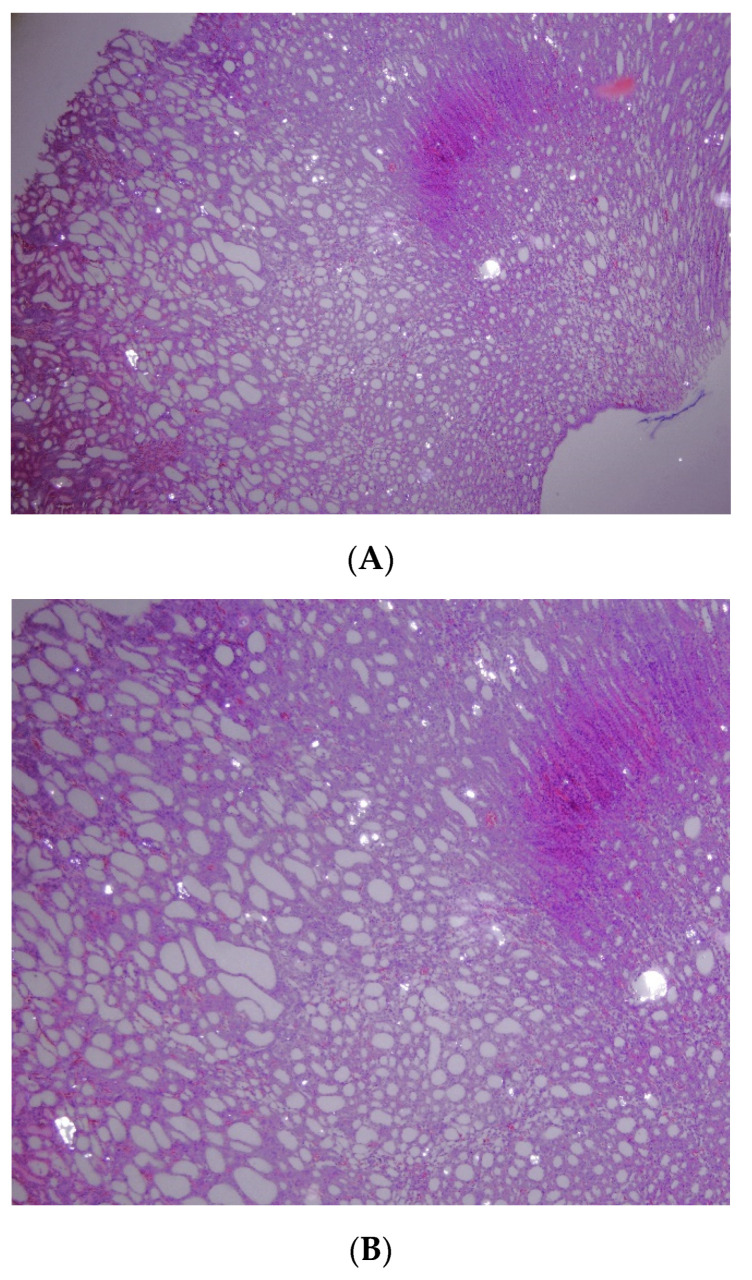
Microscopic section of a rat’s kidney reveals multiple crystal deposits in tubules ((**A**): 200×; (**B**): 400×) induced by 0.75% ethylene glycol.

The rodents we bred were housed with a light/dark cycle (light from 07:00 to 18:00) at a constant temperature (25 °C). The animal experiment and protocol should follow each country’s guidelines, and should proceed under surveillance. Rats are by far the most frequently use and most well-established animal model in stone research. This model is commonly used for hyperoxaluria and hypercalciuria in rats, due to the ease of inducing and containing CaOx crystals in the kidney, which is the most common type of human urolithiasis (Figure 1). Khan et al. induced CaOx crystals in the rats’ renal papilla tips via a single intraperitoneal injection of sodium oxalate (NaOx) [33]. They also used mini-osmotic pumps to induce crystalluria in male Sprague-Dawley rats, which was first reported in 1983 [34]. After implantation of the potassium-oxalate, the urine was found to contain abundant crystals of CaOx, hydroxyapatite, struvite and calcium phosphate. Following the injection of NaOx, CaOx microstones in the kidney and urinary bladder can be formed. Crystals in the rat’s kidney could be also observed under microscope.

Following the use of an osmotic mini-pump in the rat’s kidney, adding EG to the drinking water is more convenient as regards producing crystals in the rat’s kidney. Blood first reported CaOx calcification in the male Sprague-Dawley rat’s kidney, which was induced by a low concentration of EG (0.5% and 1.0% in diet) [35]. Lyon et al. added EG and ammonium chloride as ingestion drinks to induce CaOx crystals in the rat [36]. However, 61% of rats died within 4 weeks with ammonium chloride concentrations over 1%. Fan modified the concentration of ammonium chloride to 0.75%, and found that the formation of crystals became stable in the EG urolithiasis model [37]. However, EG alone, at concentrations between 0.50% and 0.75%, added to the drinking water remained the most common method of employment in the rat urolithiasis model [4,5,6,7,9,10,11,12,38].

Rats can also provide measurements of free radical production, via urinary enzymes and blood, in the EG induction model. Huang et al. found free radicals being produced in the early stage of EG ingestion [6]. In situ superoxide formation can also be detected in rat kidneys after nephrectomy under anesthesia [39]. Measurements of blood and urinary biochemistry were also available for rats [5,6,7,9,10,11,12,38]. Chen et al. extracted proteins from EG-induced rat kidneys, and performed 2-dimensional electrophoresis. After identification, an overexpression of alanine-glyoxylate aminotransferase-2 in rat kidneys was confirmed via proteomic study with the matrix-assisted laser desorption ionization-time of flight-mass spectrometry technique, and further confirmed by RT-PCR analysis [13]. Cherng et al. studied the relation between bacterial infection and stone disease using rats infected with proteus mirabilis for 7 days [40]. An alteration of the renal Ca^2+^-related ion channels was caused by bacteria, which down regulated monocyte chemoattractant protein-1 (MCP-1), osteopontin, and transient receptor potential vanilloid member 5. Renal inflammatory changes may be related to stone formation. Therefore, the rat model provides variable study methods for stone research.

### 3.4. Mouse

Knock-out mice provide a good model for studying genetic effects on stone formation. Mo et al. studied Tamm-Horsfall protein (THP) knock-out mice, fed with EG and vitamin D3 [41]. The results showed renal calcium crystal formation. THP has been proven to be crucial for defending nephrolithiasis formation. Salido et al. studied alanine-glyoxylate aminotransferase gene (AGAT) mutant mice, and found half of the mice developed CaOx urinary stones [42]. After administration of EG to enhance oxalate production, the mice developed renal failure and severe nephrocalcinosis. These results reveal AGAT is expressed in the liver, and is responsible for oxalate metabolism. Vernon et al. used adenine phosphoribosyltransferase (Aprt) knock-out mice to investigate the role of osteopontine (OPN) in inflammation [43]. At 12 weeks, double knock-out mice (Aprt + OPN) showed increased adenine excretion, inflammation, and crystal formation in the male mouse kidney. In female mice, the pathology change is less significant than that in male mice, except for inflammation. They concluded that OPN had the role of inflammation and crystal formation, but there were separate changes between genders. The lesser change in the female mice may be due to estrogen, which has a protective effect on calcium oxalate crystal formation [5].

There were several genetically modified mice for studying stone disease. In a Aprt knock-out mice and the 2,8-dihydroxyadenine-induced nephrolithiasis model, Tzortzaki et al. identified a decreased IMPT1 gene (an organic cation transporter), which was expressed in the kidney and contributed to impaired renal function [44]. Genetic hyperoxaluria models, such as MCP-1, may induce experimental CaOx crystalluria [45]. The knock-out of sulfate anion transporter–1 gene (Sat1; also known as Slc26a1) led to the dysfunction of oxalate transportation and hemostasis that resulted in hyperoxaluria and CaOx stone formation in mice [46]. Therefore, gene knock-out mice allow us to study a variety of oxalate metabolism-related genes. However, the gene knock-out model has the disadvantages being of high-cost, time-consuming, and limited to a single gene disease. This is the limitation of mice model.

### 3.5. Porcine

Kaplon et al. used multiparous gestation sows to test l-hydroxyproline inducing hyperoxlauria [47]. This model provides both a new animal model and a lithogenic agent. The measurement of urinary oxalate was proven to be useful in this swine model because the large amounts of urine it produces facilitates quantitation analysis. This animal model was further used by Patel et al. to compare the effects of inducing hyperoxalauria via a gelatin diet and l-hydroxyproline [48]. The results found that both agents had similar effects, but gelatin was more cost-effective. Trojan et al. tested different strategies of lithogenic agents in a porcine model [49]. They found that using 0.8% EG in the drinking water and gentamycin intramuscular injections (5 mg/kg of body weight three times a week) resulted in marked nephrotoxicity. The use of EG plus 2 µg/kg of body weight vitamin D in drinking water resulted in less nephrotoxicity and ideal crystal formations in the kidney. They concluded that EG + Vitamin D was the most cost-effective lithogenic agent [49]. Although the porcine model was proved to have some advantages, the body size appeared too huge compared with other study animals, necessitating large amounts of daily foods and more room to breed, which may limit its wide-spread use in stone research.

### 3.6. Drosophila (Fly)

The fly is an invertebrate animal, for which animal ethics are needless. There are several advantages to using flies as the study animal model for stone disease. The low cost of breeding, large number of reproductions and short life span make observations of toxicity fast, and these factors also facilitate the observation of crystal formation under polarized microscopy, reduce the amount of anesthesia needed, and reduce the space needed for breeding, making this model more common. Following Morgan et al. observing fly eye-color to prove the Mendelian law of inheritance, it is clear that the fly animal model makes it easy to reach large numbers of statistical differences [50]. It has also been easy to produce genetic manipulations in flies over the last 100 years [51,52]. Genetic manipulation is not only limited to the nervous system and behavior, but also incorporates other systemic diseases and drug discoveries [53]. Landry et al. used a fly model to study the homolog of Slc26a6 (a nephrolithiasis-related gene with the function of oxalate transportation) in terms of *Drosophila* Slc26a5/6, which found that thiosulfate decreased the CaOx stone formation in Malpighian tubules [54]. The results indicated that thiosulfate, or oxalate-mimics, may act as therapeutic competitive inhibitors for the crystallization of CaOx. Fan et al. used flies to perform RNAi knockdown of nephrolithiasis-related genes in the principal cells of Malphigian tubules, and then watched the effect of the anti-lithic agent on CaOx crystal formation [55]. Mutations of the human genes ATP6V1B1 and ATP6V0A4 have also been proven to be related to stone formation, and its homology in flies with Vha55 and Vha100-2 genes was also proved to increased crystal formation in flies [55]. Therefore, the genetic study of stone disease in flies may have future potential.

Chen et al. first observed CaOx crystals formed in Malpighian tubules after the addition of lithogenic agents to fly food within two weeks, and these were directly examined under polarized light microscopy [18]. Then, the fly model was used to screen for potential antilithic traditional Chinese medicinal plants, from 80 candidate herbs [20]. Wu et al. reported that some of the tested traditional Chinese medicine (TCM) herbs, such as *Salviae miltiorrhizae*, *Paeonia lactiflora*, *Carthami flos* and *Scutellaria baicalensis*, reached the crystal formation rate of 0.0%. However, the mechanism remains unknown, and is lacking a large, statistically significant number of animals to be further proven. Therefore, *Salviae miltiorrhizae* and *Carthami flos* underwent further studies in animal models and clinical analysis [11,20,56,57,58]. The fly model therefore quickly provides a large amount of data for screening potential antilithic drugs. Chen et al. developed a new observation method, by which a whole fly can be viewed by a micro-computerized tomography, making this model more useful [8].

Ho et al. used a fly model to test some commercial juices, such as apple, cranberry, orange and pomegranate juices, with regards to whether they were effective in preventing crystal formations in flies or not [19]. The results showed that the above commercial juices failed to prevent CaOx crystal formation. Recently, Chung et al. reported that hydroxycitric acid (HCA), an extract from *Garcinia cambogia*, can dissolve CaOx crystals in vitro [59]. This tropical fruit from Southeast Asia contains HCA, was traditionally used for cooking in India, and may be a potential anti-lithogenic herb. HCA was further studied for its preventive effect on calcium oxalate formation in the fly model [23,60].

One of the advantages of the fly model is life span study. Chemical agents potentially hazardous to human health can be rapidly tested through a life span study, because toxic agents may shorten life cycle. Flies have a short life cycle of about 8–9 weeks, and easily breed up to a large number in a short time. Therefore, the fly is an ideal model for studying potential toxic agents. Melamine and ractopamide have been reported to have potential hazardous effects on humans in Taiwan [21]. Chen et al. used a fly model to study its toxicity. Melamine significantly reduced the fly’s life span in a dose-dependent manner [17]. Ractopamide also significantly reduced life span in the fly after adding 10 ppb of ractopamide into the food [21]. Life span studies can also test any other agent with the above method.

The disadvantages of the fly model include the fact that it is difficult to study the mechanism of tubular fluid formation, and no serum or urine biochemistry can be analyzed [61]. This invertebrate animal lacks many solid organs, including kidneys. Another disadvantage of using flies is that no interventional/pharmaceutical studies can be assessed, e.g., new therapeutic medications preventing or curing stone disease, including assessment of their side effects that may occur in patients. However, the Malphigian tubules of flies have a similar genetic composition, physiology and anatomy to the human kidney [62]. Miller et al. proposed that the effects of dietary manipulation, environmental alteration and genetic variation on stone formation can be rapidly observed and quantified in flies within a few days [62].

## 4. Lithogenic Agents

### 4.1. Ethylene Glycol (EG)

EG is an agent used for antifreeze, and a raw material for the production of polyester fibers [63]. EG is the most frequent lithogenic agent, and is highly applicable in many animals, such as mice, rats and flies (different dosages may cause formation rates and intervals to be different). EG is highly water-soluble, and shows rapid gastrointestinal absorption and rapid biotransformation [64,65]. These characteristics facilitate the use of EG to induce crystal formation in animals invariable ways, such as adding into food, intraperitoneal injection or addition to drinking water. EG is successfully oxidized in the liver and turned into the more toxic compounds of glycoaldehyde, glycolic acid and oxalic acid. This will result in hyperoxaluria and CaOx crystal precipitation in animals [66]. Most researchers used EG as a typical lithogenic agent in the rodent animal model [4,5,6,7,10,11,13]. EG is stable with regards to CaOx formation after adding between 0.50% and 0.75% to drinking water. EG can also be added in the food, to induce CaOx crystal formation in the fly model [18].

### 4.2. Sodium Oxalate (NaOx)

**Figure 2:**
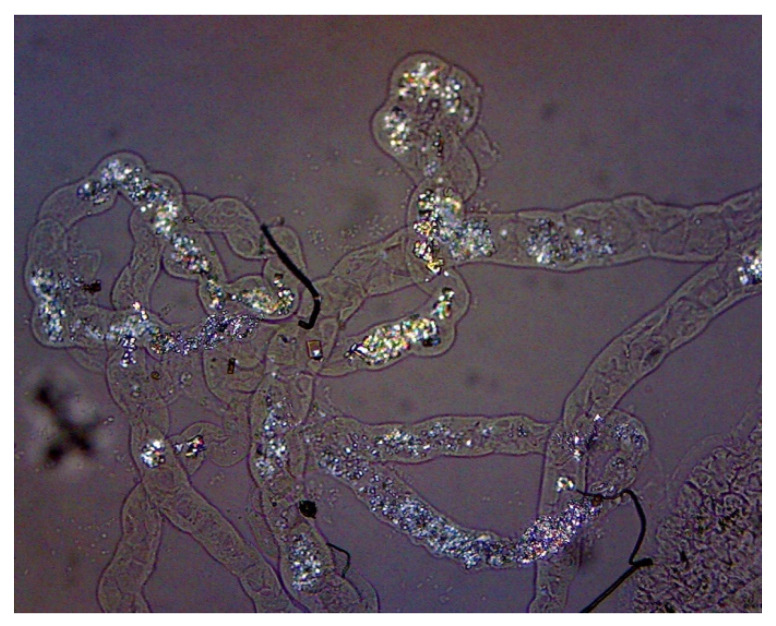
Representative view of NaOx-induced calcium oxalate crystal deposition in the Malphigian tubules of a fly (100×).

The intraperitoneal injection of NaOx into rats may cause fast crystal formation in kidneys. Crystals are formed intraluminally in the proximal tubules of the renal cortex [67]. The injection dose and interval will affect the size, number and location of crystals. A high dose of NaOx (10 mg/kg) may cause tubular necrosis and dilatation of the rat’s kidney. The oxalate excretion was five times greater than the control, and crystal presence lasted for 7 days after injection [68]. The advantage is that a single intraperitoneal injection can cause pathology change, and recovery took two weeks [33]. Crystal formation could also be induced in flies fed a NaOx diet (Figure 2).

### 4.3. l-Hydroxyproline (LHP)

LHP is a proteinogenic amino acid rich in collagen. The function of LHP is to maintain the tight collagen helix. LHP is metabolized into glyoxylate, glycolate and oxalate in the peroxisome and mitochondria of the liver [69]. A marked increase of urinary oxalate was found in rats fed with large doses of LHP. LHP was used in a sow animal model to induce renal CaOx formation [47,48]. However, in contrast with EG, LHP inducing CaOx formation in male flies’ Malphigian tubules was not universal, even after increased concentration, from 0.01% to 1.00% [18], whereas EG could induce 100% crystal formation in a concentration between 0.50% and 0.75%. Therefore, EG is a more ideal lithogenic agent than LHP in the fly model.

The other lithogenic agent was 1.5% potassium oxalate, which was added in a chow diet [45].

## 5. Preventive Agent

Potassium citrate is a typical preventive agent for CaOx stones, and is also a good positive control for animal studies. There were several reports from animal studies regarding its antilithic effect. Tested herbal medicine, such as Wulingsan, Zhulingtang and *Salviae miltiorrhizae*, had been proven to have a potential antilithic effect, but this necessitates further clinical application to prove [7,10,11,56,70]. Wulingsan had been trialed clinically in a short period, and had a diuretic effect without interfering electrolytes [71]. However, a nation-wide population study of Wulingsan did not find its preventive effect clinically [70]. HCA has proven its antilithic effect both in vivo and in vitro [23,59,60]. However, further clinical trials for proving HCA’s effect are needed.

## 6. Future Perspective

Table 1 depicts the advantages and disadvantages of several animal models. All lithogenic agents are not normally oral food for humans. The fly is an invertebrate animal lacking real kidneys, liver, lungs, etc. Rodents are popular experimental animals for a variety of research. However, rodents’ nocturnal animal behavior is unlike that of humans. Dogs and pigs are relatively larger animal for study, for which the breeding costs are high. There is no ideal animal model for studying urolithiasis. We recommend fly as a future study animal, on account of its many advantageous characteristics. Genetic studies of flies help identify potential candidate genes responsible for stone disease [72]. Cohen et al. support using the *Drosophila* excretory system in order to study many human diseases [73]. They reviewed the anatomy and physiology of the fly’s Malphigian tubule, and proposed that the fly is an excellent model for studying many renal functions, renal stone diseases and cancer-promoting processes. Therefore, the fly model has wide future applicability.

## 7. Conclusions

There were several animal models useful for the study of stone disease, including rat, mouse, fly, dog and porcine. EG currently seems to be an ideal lithogenic agent. The fly model seems to have future prospective use in studying human stone disease, due to its many advantages, such as low cost, high numbers, and its allowing of genetic studies.

## Figures and Tables

**Table 1:** Comparisons of advantages and disadvantages in variable animal models.

Animal	Fly	Rat	Mouse	Pig	Dog
Cost	Low	Intermediate	High	Low	High
Research organ	Malphigian tubules	Kidney	Kidney	Ureter	Kidney
Preparation of crystal observation	Direct observe under Polarizing microscopy	H&E stain before microscopy	H&E stain before microscopy	H&E stain before microscopy	H&E stain before microscopy
Biochemical measurement	Not available	Available	Available	Available	Available
Lithogenic agent	EG, LHP,	EG, NaOx	EG, NaOx	EG+VD, LHP	Not available
Requirement of animal ethic	-	Yes	Yes	-	Highly recommended

EG: ethylene glycol, NaOx: sodium oxalate, LHP: l-hydroxyproline, VD: vitamin D.
